# Perceived cervical cancer risk among women treated for high-grade cervical intraepithelial neoplasia: The importance of specific knowledge

**DOI:** 10.1371/journal.pone.0190156

**Published:** 2017-12-22

**Authors:** Sonia Andersson, Karen Belkić, Selin Safer Demirbüker, Miriam Mints, Ellinor Östensson

**Affiliations:** 1 Department of Children’s and Women’s Health, Division of Obstetrics and Gynecology, Karolinska University Hospital-Solna, Karolinska Institutet, Stockholm, Sweden; 2 Department of Oncology-Pathology, Karolinska Institute, Stockholm, Sweden; 3 School of Community and Global Health, Claremont Graduate University, Claremont, California, United States of America; 4 Institute for Prevention Research, Keck School of Medicine, University of Southern California, Alhambra, California, United States of America; 5 Department of Medical Epidemiology and Biostatistics, Karolinska Institutet, Stockholm, Sweden; Rudjer Boskovic Institute, CROATIA

## Abstract

**Objective:**

Women with high-grade cervical intraepithelial neoplasia (CIN) are at increased risk for developing cervical cancer. We examine how women with high-grade CIN perceive their own risk, and about pertinent knowledge concerning human high-risk papillomavirus (HPV), CIN and cervical cancer.

**Methods:**

All patients who underwent first-time treatment of high-grade CIN (grade 2+) were followed-up at 6-months at the Karolinska University Hospital, Stockholm, Sweden and were invited to participate in the present study. This included completion of a questionnaire examining sociodemographic characteristics, self-perceived risk of cervical cancer without regular gynecologic follow-up, and 14 queries about HPV, CIN and cervical cancer knowledge, inter alia.

**Results:**

The participation rate was 96.6%, with 479 women enrolled in this study. Over 75% were age 40 or younger, over half had completed university education. Most were married or co-living with their partner and were gainfully employed. On a scale scored from 10 (highest self-perceived risk of cervical cancer without regular gynecologic follow-up) to 1 (lowest self-perceived risk), 64% rated their risk ≥ 7; almost 30% viewed their risk ≤ 6 and 7.5% did not rate their risk. A Specific Knowledge Scale with six of the queries explained 58.3% of the total variance. Nearly 30% of the women answered four or fewer of the six queries correctly. The Specific Knowledge Scale predicted self-perceived cervical cancer risk (Odds ratio = 11.3, 95% Confidence Interval 5.6 − 22.6) after adjusting for age, income and education. Most of the women with low self-perceived cervical cancer risk did not rate their HPV-related knowledge as good. However, 32 predominantly university-educated women, with low self-perceived cervical cancer risk, considered their HPV-related knowledge good.

**Conclusion:**

It is vital to effectively convey accurate information about these patients’ cervical cancer risk, needed preventive and follow-up measures, together with the relevant specific knowledge, for these women at increased risk for developing cervical cancer. Tailored programming to address these knowledge gaps is needed.

## Introduction

Among women worldwide, cervical cancer is the fourth most common cause of cancer death [1]. An estimated 265,700 women died in 2012 alone due to cervical cancer. The vast majority of these women were from less developed regions of the world [2]. Cervical cancer has been aptly termed “a disease of disparity”, with these disparities in incidence and mortality readily apparent in Europe [3]. Within the European Union (EU), the highest cervical cancer mortality is seen in those countries in which participation in screening programs is the lowest [3].

Whenever in place, high participation in screening programs through the Papanicolaou test (cytology-based) and subsequent treatment of cervical dysplasia has effectively reduced the cervical cancer mortality [4–6]. An invitational, population-based cytology-based cervical cancer screening program has been in place in Sweden since 1967, with a participation rate of 73% (as of 2010), and cervical cancer mortality rate the 9^th^ lowest in the EU [3, 7]. Nevertheless, nearly 500 women are diagnosed annually with cervical cancer and approximately 200 women per year die from the disease in Sweden [8, 9]. Cervical cancer is considered a major public health issue in Sweden, with a heavy economic burden, estimated as €58 million in 2006 alone [10].

The identification of high-risk human papilloma virus (HPV) as the key etiologic factor for cervical cancer is impacting upon approaches to screening and prevention of this malignancy. Testing for HPV is more sensitive, although often less specific than cytology-based testing for detection of high-grade cervical intraepithelial neoplasia (CIN) [1, 5]. Vaccination against HPV has successfully reduced the proportion of abnormal cytology screening tests, with a decrease in the subsequent need for diagnostic colposcopy [11]. Nevertheless, recommended screening guidelines are not modified on the basis of HPV vaccination [6].

These considerations are particularly germane for women in whom high-grade CIN has been detected and treated. In a study of 330 women with cervical dysplasia treated with the loop electrical excision procedure (LEEP), a negative high-risk HPV test plus negative cytology at six months were reported to be a reliable test of cure at three-year follow-up [4]. On the other hand, a population-based investigation indicates that women with grade 3 CIN are at long-term increased risk of HPV-related carcinomas of the anogenital and oropharyngeal regions, thus warranting even more intensive screening and other measures [12]. Moreover, a Swedish population-based registry study indicates that women treated for grade 3 CIN are at elevated risk of developing and dying from cervical or vaginal cancer, compared with the general female population. The risk was found to increase after age 60 years, indicating that lifelong surveillance is needed [13].

In a study from our group of ninety patients followed-up after treatment for CIN with conization via LEEP using a C-LETZ electrode, the presence of high-risk HPV genotype predicted all five cases of residual high-grade squamous intraepithelial lesion/CIN 2+, and the lack of persistent high-risk HPV infection was the most specific indicator of absence of recurrent or residual high-grade disease [14]. A subsequent follow-up study of 149 patients showed that high-risk HPV predicted all cases of treatment failure among the women with high-grade disease [15]. Moreover, a recent 5-year longitudinal investigation from Northern Italy [16] of 310 patients with CIN2+ indicates that none of the 172 women with a negative HPV DNA test at six-months post-treatment had residual or recurrent CIN2+ during the two-year surveillance period. Thus, the authors conclude that that HPV DNA is highly predictive of disease eradication.

Especially in light of these new and unfolding developments, accurate risk assessment and information concerning HPV and recommended screening follow-up are essential. Women with high grade CIN warrant special attention in this regard. To the best of our awareness, however, knowledge and perception of risk have not been previously investigated in this group of women. The aim of the present study is to examine self-perceived risk of cervical cancer and pertinent knowledge about HPV, CIN and cervical cancer among women treated for high-grade CIN. We will also assess relevant sociodemographic characteristics, HPV vaccination status as well as performing needs assessment regarding how the relevant knowledge could best be conveyed. It is hoped that these efforts will contribute to improving adherence to necessary follow-up among women at increased risk for developing cervical cancer.

## Methods

### Participants in the study

Patients who had undergone first time treatment of high-grade CIN (grade 2 or higher) and attended the first follow-up 6 months thereafter at the Karolinska University Hospital, Stockholm, Sweden, were the target group for the present study. Each patient received a written invitation letter via postal mail. Brief salient facts about HPV and cervical cancer were included in the invitation letter, as well as contact information for the Attending Gynecologist and Principal Investigator (SA) and for the Research Coordinator (EÖ). The study was presented as entailing completion of a questionnaire with the aim of helping to better prevent cervical cancer plus an HPV self-test. Complete confidentiality and freedom to withdraw from the study at any time without any adverse consequences whatsoever were guaranteed.

The clinical research team ensured that the scheduled follow-up appointment was convenient for each patient. With this concerted effort, all the patients who had been first-time treated for high-grade CIN attended 6-month follow-up.

At the 6-month follow-up visit the Research Coordinator (EÖ) met with each patient to explain the study procedure. All invited women signed an informed consent form with the choices of agreeing or declining to participate in the study. Approval of the study protocol was obtained by the Ethical Review Board at Karolinska Institute, Stockholm, Sweden (reference numbers 2006/1273-31, 2014/2034-32).

Knowledge of the Swedish language was a requirement for participation in the questionnaire portion of the study, since the questionnaire was in Swedish. A total of 480 women were eligible to participate in the questionnaire portion of the study, of whom 479 signed the informed consent in agreement and were thus included in the present study. In addition, sixteen women who would have otherwise been eligible (i.e. first-time treated for high-grade CIN and attending 6-month follow-up), did not complete the questionnaire due to lack of fluency in the Swedish language. Thus, the actual participation rate was 96.6%. The mean age of the seventeen non-participants was 42 ± 7.8.

### Questionnaire

The questionnaire examined socio-demographic characteristics such as age, civil status, personal annual income, education and employment status. Queries were included concerning knowledge about HPV, CIN and risk of cervical cancer. These knowledge queries were similar, though not identical to those used in a previous study among women attending clinic-based cervical cancer screening in Stockholm [17]. For each of the 14 statements there were three options: endorsement, disagreement and that the participant does not know whether or not the statement is correct. The participant was then asked whether she considered her knowledge about HPV good, with the same three options: endorsement, disagreement and does not know. Thereafter, a list of possible means by which the participant would like to receive more information about HPV, cervical cancer and its prevention was presented. The options were through 1) health professionals (mid-wives, gynecologists, primary care physician), 2) educational programs on television or radio, 3) health education through work or school, 4) informational brochures sent by postal mail, 5) through the internet or 6) other means. All preferred options could be marked. In part 4 the participant was asked whether or not she had been vaccinated for HPV (the “does not know” option was also provided). If affirmative, the age of vaccination was queried. The participant was then asked to rate on a scale from 10 to 1, with 10 being the highest and 1 being lowest, how she perceives her own risk of developing cervical cancer without regular gynecologic follow-up. The participant was subsequently queried about how often she thought that gynecologic follow up should be performed for her to avoid developing cervical cancer later in life.

### Statistical analysis

Thorough univariate analysis was done, with the distribution of all continuous and semi-continuous variables assessed visually, as well as by examination of skewness and kurtosis. Parametric bivariate analysis was performed insofar as the continuous or semi-continuous variables had both skewness and kurtosis < 1, otherwise non-parametric bivariate analysis was carried out. For bivariate analysis of dichotomous variables, Yates chi-squared analysis was used. Factor analysis was employed to develop the Specific Knowledge Scale, as described in the Results section. Unless otherwise noted, an absolute value of the factor loading > 0.70 was required for inclusion of a component in the Specific Knowledge scale. Binomial logistic regression was performed to compute odds ratios (OR) and 95% confidence intervals (CI) for unadjusted and adjusted models. All statistical analysis was carried out with Statistica 64 software.

## Results

### Univariate findings

The socio-demographic characteristics of the cohort are presented in Table 1. Therein, it is seen that over 75% of the participants in the study were age 40 or younger. The majority had a personal gross annual income of 360 000 Swedish kronor (SEK) or more (~ $42 000), with nearly 25% earning over 800 000 SEK (~ $93 400) ($1 = 8.5613 SEK year 2016). Over half of the participants had completed university education. Well over half of the participants were either married or living with their partner. More than 75% were gainfully employed.

**Table 1:** Socio-demographic characteristics of the patients with high-grade CIN.

	N	Percentage
**Age** (years)		
18 to 30	177	37.1
31 to 40	187	39.2
41 to 50	83	17.4
51 to 60	23	4.8
61 and over	7	1.5
Missing	2	
**Gross annual income** (SEK)*		
Up to 99 0000	55	11.9
100 000 to 259 000	51	11.0
260 000 to 359 000	101	21.9
360 000 to 799 000	147	31.8
≥ 800 000	108	23.4
Missing	17	
**Education** (highest completed)		
Elementary school	25	5.2
Gymnasium	175	36.6
University	278	58.2
Missing	1	
**Civil status**		
Married	142	29.8
Co-living	185	38.8
Has partner, but lives apart	43	9.0
Single	105	22.0
Widow	2	0.4
Missing	2	
Main activities**employment status		
Gainfully employed (paid employment)	378	78.9
Self-employed	33	6.9
Student	44	9.2
Seeking work	14	2.9
Disabled/retired	24	5.0
Other main activity	10	2.1

*$1 = 8.5613 SEK year 2016

**More than one option is possible for the query about main activities

Altogether 42 of the participants reported having been vaccinated. Twenty-four stated that had done so at age 21 or above, fourteen reported having been vaccinated between the ages of fifteen and twenty, and four women did not know or did not answer the query about age at vaccination. Although 395 (82.5%) of the participants stated that they knew that they had not been vaccinated, seventeen women stated that they did not know their vaccination status, and twenty-five did not answer the query about HPV vaccination.

Perceptions about risk of developing cervical cancer together with queries concerning knowledge about HPV, CIN and cervical cancer are summarized in Table 2. Altogether 63.7% of the participants considered their risk to be 7 or more on the scale of 10; whereas almost 30% viewed their risk as moderate to low and nearly 8% did not answer this query. About 85% of the women considered that gynecologic follow-up was needed biennially or annually to protect themselves from developing cervical cancer. The percentage of correct answers to the 14 queries assessing knowledge about HPV, CIN and cervical cancer varied substantially. Only two questions were correctly endorsed by over 80% of the participants; namely, that HPV exists and that cellular changes over a longer period can lead to cervical cancer. The lowest percentage of correct answers (13.2%) was that some types of HPV can lead to other cancers in both women and men. Fewer than half of the participants endorsed that HPV has various types, that both men and women can be infected more than once in their lives, that HPV is most often found in young people, but can be found in all age groups and that HPV can lead to condylomata. Altogether barely 30% of the women considered their knowledge about HPV to be good.

**Table 2:** Risk perception and knowledge about HPV, CIN and cervical cancer among the patients with high-grade CIN.

	N	Percentage
**Self-assessed risk of developing cervical cancer without regular gynecologic follow-up** (1 = lowest, 10 = highest)		
9 to 10	123	25.7
7 to 8	182	38.0
5 to 6	104	21.7
3 to 4	31	6.5
1 to 2	3	0.6
Did not answer	36	7.5
**Perceived frequency of gynecologic follow-up needed to protect this patient, herself, from developing cervical cancer**		
Annually	236	49.3
Biennially	172	35.9
Triennially	31	6.5
Every fourth year	12	2.5
Never	0	
Did not answer	28	5.8
**Endorses that:**		
**There is a virus which is called human papilloma virus (HPV)**		
**Yes**	**394**	**82.2**
No	23	4.8
Did not know	45	9.4
Did not answer	17	3.6
**HPV has various types**		
**Yes**	**204**	**42.6**
No	71	14.8
Did not know	184	38.4
Did not answer	20	4.2
**HPV is sexually transmitted between partners**		
**Yes**	**342**	**71.4**
No	50	10.4
Did not know	67	14.0
Did not answer	20	4.2
**Both men and women can be HPV infected at one or more times in their lives**		
**Yes**	**236**	**49.2**
No	92	19.2
Did not know	132	27.6
Did not answer	19	4.0
**HPV is most frequently found in young people, but can occur in all age groups**		
**Yes**	**204**	**42.6**
No	85	17.7
Did not know	171	35.7
Did not answer	19	4.0
**Women and men can be infected with HPV without having any symptoms**		
**Yes**	**354**	**73.9**
No	31	6.5
Did not know	75	15.6
Did not answer	19	4.0
**In most cases, HPV clears by itself**		
**Yes**	**271**	**56.6**
No	72	15.0
Doesn’t know	117	24.4
Did not answer	19	4.0
**A prolonged HPV infection can in some cases can be the source of cellular changes in the uterine cervix**		
**Yes**	**377**	**78.7**
No	32	6.7
Did not know	51	10.6
Did not answer	19	4.0
**Cellular changes can over a longer period lead to cervical cancer**		
**Yes**	**417**	**87.0**
No	14	2.9
Did not know	29	6.1
Did not answer	19	4.0
**Some types of HPV can lead to other cancers in both women and men**		
**Yes**	**63**	**13.2**
No	108	22.5
Did not know	286	59.7
Did not answer	22	4.6
**Some types of HPV can lead to genital warts, so called condyloma**		
**Yes**	**167**	**34.9**
No	81	16.9
Did not know	211	44.0
Did not answer	20	4.2
**Vaccination is one way to protect oneself against HPV infections that can lead to cell changes and in some cases to cervical cancer**		
**Yes**	**381**	**79.5**
No	19	4.0
Did not know	59	12.3
Did not answer	20	4.2
**The vaccine is most effective if given prior to sexual debut**		
**Yes**	**311**	**64.9**
No	31	6.5
Did not know	118	24.6
Did not answer	19	4.0
**It is important to continue with gynecological check-ups even if one is vaccinated, since vaccination does not provide full protection**		
**Yes**	**338**	**70.6**
No	27	5.6
Did not know	95	19.8
Did not answer	19	4.0
**“I think that I have good knowledge about HPV”**		
**Yes**	**144**	**30.1**
No	218	45.5
Did not know	95	19.8
Did not answer	22	4.6

### Construction of the Specific Knowledge Scale

**Fig 1:**
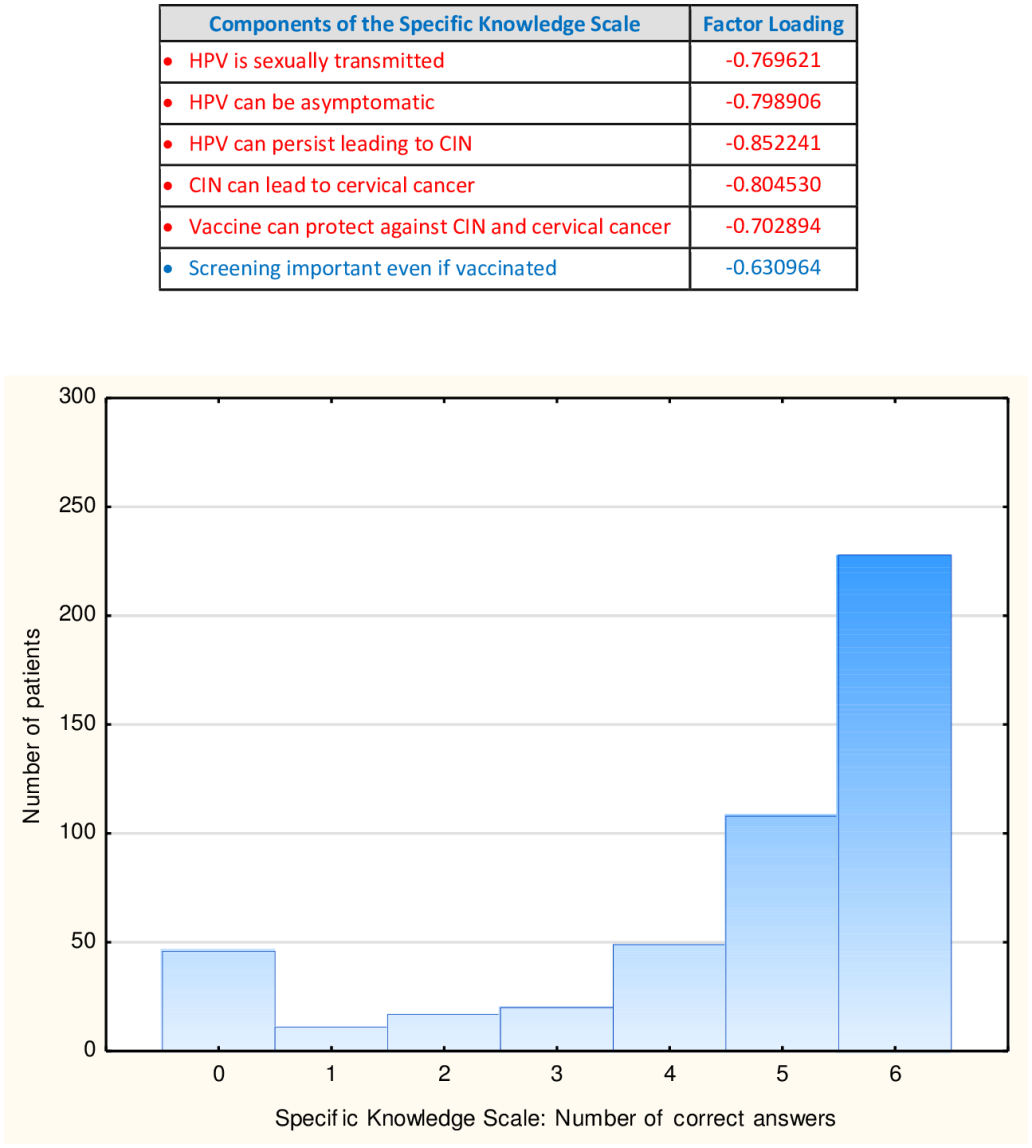
The Specific Knowledge Scale. The upper panel displays the factor loadings of the six queries included in the Specific Knowledge Scale. In the lower panel are the number of correct answers to the Specific Knowledge queries along the abscissa and the number of patients along the ordinate, where the total number of patients in the present study is 479.

Factor analysis of the 14 knowledge queries concerning HPV, CIN and cervical cancer yielded two factors: one with an Eigenvalue of 6.3, explaining 45.3% of the variance, and the other with an Eigenvalue of 1.2, explaining 8.4% of the variance. Five of the queries had an absolute value of their factor loading above 0.70. These are marked in red in the upper panel of Fig 1.

We considered these queries to be not only psychometrically suitable, but also to be the most salient from the clinical and public health vantage points. With the latter in mind, we included one additional query whose absolute value of its factor loading was 0.63, namely: Screening is important even if vaccinated. The newly created Specific Knowledge Scale with these 6 factors had an Eigenvalue = 3.5 and explained 58.3% of the total variance. The lower portion of Fig 1 graphically displays the number of patients along the ordinate, with the abscissa being the number of correct answers on the Specific Knowledge Scale. Forty six of the participants (nearly 10%) did not answer any of the 6 queries correctly, and one hundred forty three (nearly 30%) answered 4 or fewer of the queries correctly.

### Salient significant bivariate findings

Age and annual income were correlated (Pearson r = 0.25, p = 0.000), such that the youngest women had the lowest personal gross annual income. The patients who were co-living were younger (mean age = 32.2 ± 7.1), whereas those who were married were significantly older (mean age = 38.1 ± 7.8), (2-sample “t” tests, both with p = 0.0000). The youngest women also had the lowest self-assessed risk of developing cervical cancer without regular gynecologic follow-up (Pearson r = 0.147, p = 0.002). There was an inverse correlation between age and Specific Knowledge Scale score (Spearman *ρ* = −0.095, p = 0.039), indicating that the younger women had higher specific knowledge.

There was a positive correlation between personal gross annual income and educational level (Pearson r = 0.29, p = 0.000), indicating that the women with higher income had a higher level of completed education. The married women also had higher personal annual income (2-sample “t” test, p = 0.03). Annual income was positively correlated with self-assessed risk of developing cervical cancer without regular gynecologic follow up (Pearson r = 0.12, p = 0.012). Thus, the participants with lower income perceived themselves to be at lower risk of cervical cancer. There was a direct correlation between annual income and Specific Knowledge Scale score (Spearman *ρ* = 0.14, p = 0.003); the women with lower income thus had lower specific knowledge about HPV, CIN and cervical cancer.

The participants with higher completed education were more often co-living, whereas those with less completed education were more often single (2-sample “t” tests, both with p = 0.01). Education and Specific Knowledge Scale score were directly correlated (Spearman *ρ* = 0.22, p = 0.000); the women with lower completed education had lower specific knowledge about HPV, CIN and cervical cancer. The women who did not know that HPV can lead to other cancers had a lower educational level (post-hoc Bonferroni p = 0.03) compared to those who endorsed this statement (one-way analysis of variance (ANOVA), F = 3.16, p = 0.02).

Perceived frequency of gynecologic follow-up needed to protect the patient from developing cervical cancer was correlated with self-assessed risk of developing cervical cancer without regular gynecologic follow up (Spearman *ρ* = 0.21, p = 0.00001). Concordantly, the Specific Knowledge Scale score was correlated with perceived frequency of needed gynecologic follow up (Spearman *ρ* = 0.11, p = 0.02). The women who considered their own knowledge good had higher scores on the Specific knowledge scale (2-sample “t” test, p = 0.0000).

One-way ANOVA with post-hoc Bonferroni probabilities reveal that the women who stated that they had been HPV vaccinated were younger (mean age = 27.4 years) than those who stated that they were not vaccinated (mean age = 35.6 years) (p = 0.000). The women who did not answer the query about HPV vaccination were significantly older (mean age = 40.3 years) than each of the other groups of women. The women who did not know their HPV vaccination status had a mean age of 32.9 years.

More significant bivariate findings will be reported in the subsection on Needs Assessment. Further analysis of self-assessed risk of developing cervical cancer will be presented in the next subsection.

### Outcome variable: Perceived risk of cervical cancer without regular gynecologic follow-up

The variable: self-assessed risk of developing cervical cancer without regular gynecologic follow up was dichotomized at 7 or greater (realistic) versus 6 or less. The thirty-six patients who left this query blank were placed in the latter category, i.e. as not indicating a realistic assessment of their own risk. Table 3 presents the unadjusted binomial logistic regression model with realistic risk assessment as the dependent variable, and Specific Knowledge score as the independent variable. Therein, the OR was over 12, with the 95% CI ranging from just above 6 to over 23. This model included all 479 participants in the study.

**Table 3:** Realistically assessed risk of cervical cancer without regular gynecologic follow up: Relation to objectively-assessed specific knowledge and self-assessed knowledge among patients with high-grade CIN.

	Specific Knowledge Score
**OR**	**12.15166**
-95% CI	6.280282
+95% CI	23.51215

Model chi-squared = 65.6, p = 0.0000, N = 479

The adjusted binomial logistic regression model is presented in Table 4. Therein, age, annual income and education were included, together with Specific Knowledge score, as the independent variables. The OR’s, although greater than one, were not significant for any of the former variables. Adjusting for these 3 covariates, the Specific Knowledge score retained its very high OR, with only a slight reduction to ~ 11.3, and ± 95% CI of similar width (~17). This multi-variate model included nineteen fewer patients, due to the missing data for age, income and education.

**Table 4:** Logistic regression model for realistic risk assessment, adjusted for age, income and education.

	Age	Annual Income	Education	Specific Knowledge Score
**OR**	2.055215	1.610231	1.349815	**11.27492**
-95% CI	0.7012011	0.6897754	0.6432917	5.631041
+95% CI	6.023821	3.758966	2.832308	22.57555
p values	0.188	0.27	0.43	p = 0.000

Model chi-squared = 66.6, p = 0.0000, N = 460

Table 5 presents the 2 x 2 frequency table for realistic risk assessment versus good self-assessed knowledge about HPV. Yates’ chi-squared analysis reveals that significantly more than the expected number of women with realistic risk assessment also considered their HPV knowledge to be good. Conversely, significantly more than the expected number of women who underestimated their risk of developing cervical cancer without regular gynecologic follow-up did not endorse that they considered HPV knowledge was good. Albeit fewer in number than expected from the Yates’ chi-squared analysis, there were thirty two study participants who underestimated their cervical cancer risk but, nevertheless, considered their HPV knowledge to be good. The latter two groups will be a particular focus of the analysis of the next sub-section.

**Table 5:** Yates chi-squared analysis of the relation between self-assessed HPV knowledge and realistic assessment of cervical cancer without regular gynecologic follow-up.

	Not yes: Good self-assessed HPV knowledge	Good self-assessed/HPV knowledge
Risk not realistically assessed (< 7 or unstated)	142	32
Realistic risk assessment (≥ 7)	193	112

Yates chi-squared = 16.85, p = 0.0004, N = 479

### Needs assessment

As seen in the left column of Table 6, over two-thirds of the participants stated that they would like to receive more information about HPV, cervical cancer and its prevention from health professionals (midwives, gynecologists, primary care physicians). This was, by far, the most frequently chosen option. The next most frequently chosen option was through the internet, followed by postal mail, then from work or school, with television or radio being the least frequent explicit option chosen by the entire cohort. The subgroups of women who did not realistically assess their risk of cervical cancer without regular gynecologic follow-up are also examined further. Namely, in the middle column of Table 6 are the data for the 142 women who did not endorse that their HPV-related knowledge was good and who also did not realistically assess their cervical cancer risk. In the right column are the data for the 32 women who considered their HPV-related knowledge was good but did not realistically assess their cervical cancer risk. These latter two groups of women, whose mean age was identical (34.3 years), did not differ significantly in their desired ways of acquiring more information about HPV, cervical cancer and its prevention. However, the second most frequently chosen option was through the internet for those who did not endorse that their HPV knowledge was good. On the other hand, for those who considered their HPV knowledge good, post mail was the second most frequently chosen option. The women who considered their HPV knowledge good despite lack of realistic risk assessment had significantly more often completed university education compared to the women who did not realistically assess their risk but did not endorse that their HPV knowledge was good.

**Table 6:** Needs assessment for women with high grade CIN: Focus on those with low perceived cervical cancer risk.

Wants more information from:	Entire cohort with high grade CIN (N = 479)		Not realistic perceived cervical cancer risk & Not yes to good self-assessed HPV-related knowledge (N = 142)			Not realistic perceived cervical cancer risk & Yes to good self-assessed HPV-related knowledge (N = 32)	
	N	Percent	N	Percent		N	Percent
Health professionals	327	68.3	91	64.1		22	68.8
TV/radio	68	14.2	21	14.8		5	15.6
Work/school	119	24.8	30	21.1		4	12.5
Via postal mail	172	35.9	44	31		13	40.6
Internet	179	37.7	60	42.3		9	28.1
Other sources	7	1.5	1	0.7		1	3.1
University Degree	278	58	66	46.5	*	22	68.8
	Mean	Sd	Mean	Sd		Mean	Sd
Age	35.0	8.8	34.3	9.2		34.3	9.3

*Yates chi-squared 4.3, p = 0.038

The participants who would like more information from health professionals and from work or school were younger (2-sample “t” tests, both with p = 0.002). On the other hand, women who stated that they would like more information from television or radio, or via postal mail had lower incomes (2-sample “t” tests, p = 0.001 and p = 0.048, respectively). Women with lower completed education also stated that they would like more information from television or radio (2-sample “t” test, p = 0.03).

## Discussion

The most robust, strategically and clinically important finding of the present study is that specific knowledge about HPV, CIN and cervical cancer powerfully predicts realistic self-assessment of cervical cancer risk among this at-risk cohort. This finding is independent of age, income and education, the former two of which are also significantly associated with realistic risk assessment. Unrealistic optimism has been identified as an important predictor of lack of intention to participate in early detection programs for cervical cancer and other malignancies [18]. The danger of unrealistic optimism is far greater in the present cohort due to the high risk of developing cervical cancer insofar as appropriate follow-up is not carried out. Since specific knowledge about HPV, CIN and cervical cancer can be readily enhanced, a focal point for intervention efforts is hereby clearly identified.

By far the most preferred modality, as indicated by the participants themselves, is that further information be provided by health professionals. This finding suggests that time and resources should be set aside for the health care providers to ensure that all women with high-grade CIN have acquired the needed knowledge to realistically assess their own risk status. This is expected to critically contribute to full adherence to recommendations for further needed gynecologic follow-up. An atmosphere of confidence and trust is the vital precondition to frame the risk assessment in an empowering manner, through objective knowledge, and avoiding the distress that has been reported in association with colposcopy and subsequent HPV testing [19, 20].

The very high participation rate in the present study suggests that such a secure atmosphere was established. The active involvement of the clinical-research team in personally presenting the study to all the eligible women appears to have been very effective. Coherently, in various settings it has been shown that personal contact helps promote women’s enrollment in studies of early cancer detection and prevention [21, 22].

Notwithstanding the nearly complete participation of the invited women, their knowledge about HPV, CIN and cervical cancer risk was overall quite low. This is of major concern and quite surprising given that these women had been screened, visited a gynecologist, had been treated and then followed-up. Nevertheless, they were still not sufficiently informed about the HPV, CIN and cervical cancer. The opportunities to provide information during these visits within the health care system appear to have been very often missed.

It is also noteworthy that awareness of the risk for both women and men of developing other HPV-related cancers was the lowest of all the fourteen knowledge queries. Fewer than fifteen percent of the participants endorsed this statement. This small group of women who did endorse the statement were those with higher education compared to the vast majority of the women did not know the answer to this query. This knowledge is particularly important for women with high-grade CIN, for whom, as noted, more intensive follow-up and other measures are recommended due to long-term increased risk of HPV-related carcinomas of the anogenital and orpharyngeal regions [12]. A similarly low level of knowledge about the risk of other HPV-related cancers was reported in a cross-sectional study of U.S. adults [23]. In that study, the response rate was 34.4%, such that presumably in the general population, awareness of other HPV-related cancers is even lower. Especially since precancerous lesions of the ano-genital region can be identified and treated with excision or ablation (the current standard of care) [24], there is a clear need for more widespread information dissemination about other HPV related cancers in both men and women.

This knowledge is also of general public health importance in promoting adherence to HPV vaccination for both genders. In a recent Swedish study, knowledge about the benefits of HPV vaccination for boys was reportedly low among parents who were offered HPV vaccination for their pre-teenage daughters [25]. In the present study, a number of further salient knowledge gaps were noted in relation to HPV vaccination. Namely, over twenty percent of the women were not aware of the protective role of HPV vaccination. Moreover, nearly ten percent of the women either did not know their own vaccination status or did not answer the query.

Among the women who did not appear to have a realistic assessment of their cervical cancer risk, two subgroups were identified. The first, and larger, subgroup was comprised of those did not consider themselves to have good HPV related knowledge. Fewer of these women had completed a university education. In a study from the Appalachian region of the U.S., women referred to colposcopy after abnormal Pap smears were frequently found to have limited understanding of these findings. It was only the women with high health literacy skills who accurately grasped the meaning of these findings. The authors suggested that counseling should be tailored accordingly during the clinical encounter [26]. Such a strategy would seem to be appropriate for this sub-group of women in the present study. Thus, focused attention is needed to enhance specific knowledge about HPV, CIN and cervical cancer, among women with high-grade CIN whose level of education is lower. The potential contribution of the media, i.e. television and radio, in conveying relevant information about HPV and screening for cervical cancer was also indicated among the women with lower education and lower income in the present study. Many such effective media-based programs have been carried out targeting these populations [27–29], although perception of risk remains difficult to affect [28].

In contrast, however, we identified a subgroup of women, albeit small, who were generally highly educated, considered their HPV-knowledge to be good, but did not realistically identify their own elevated risk of developing cervical cancer without regular gynecologic follow-up. Underestimation of one’s own cervical risk has been described among some university educated women in the U.S. [30, 31]. On the other hand, there is also a report that university educated women diagnosed with HPV were likely to consider themselves at risk for developing cervical cancer [32]. The role of health professionals has been underscored for this group of women, emphasizing the critical importance of accurately conveying the HPV-related knowledge to university-educated women [30].

Direct communication by postal mail may be a helpful modality, and was noted to be one of the preferred options, not only among this subgroup of women, but also for the entire cohort. Postal mail communication was also more often chosen among women with lower incomes. An early study on the use of mailed letters that contained tailored, personalized messages based upon medical records showed this strategy to be particularly effective in promoting cervical as well as breast cancer screening among women with low incomes, as well as those from minority backgrounds [33].

It has been noted that in most countries with cervical cancer screening programs, the majority of cervical cancers occur in women who have not been regularly screened [34]. Low income and low levels of education have frequently been associated with non-participation in cervical cancer screening [35–37], although the evidence is not entirely consistent [38]. Among women aged 20 to 64 in 2014 in Sweden, median annual gross income is approximately 280 000 Swedish kronor (~ $32 700 USD), with over 90% of these women earning less than 450 000 (~ $52 500 USD) [39]. Thus, the present cohort has a substantially higher income with nearly 25% earning more than 800 000 kronor per year (~ $93 400 USD). Concordantly, while nearly 60% of the present cohort reported having completed university education, only 39% of the women age 25 to 44 in Sweden had done so, as of 2015 [39]. On the other hand, 9% of the women age 25 to 44 in Sweden had only compulsory education, while this was the case for 5.2% of the study cohort [39].

The age-adjusted incidence of cervical cancer in Sweden is not reported to differ among persons born outside Sweden and those born in the country [40]. With regard to cervical cancer mortality, although the data are not entirely consistent [40], the cancer mortality rate ratio, adjusted for age at follow-up for the period 1961 to 2009, was reportedly higher (1.22, 95% CI 1.14 − 1.32) for women born outside Sweden compared to those who were Swedish born [8]. There are many reports of lower attendance to cervical cancer screening programs among women of ethnic minority backgrounds and/or born outside the host country [36, 41–44]. A population-based study from Stockholm examining the period 1994 to 1996 did not find lower participation in invitational cervical cancer screening among women born outside Sweden compared to those who were born in Sweden [45]. However, a more recent investigation encompassing all of Sweden from 1993 to 2005 indicates that the degree of participation in cervical cancer screening was significantly lower (49%) among immigrant women versus 62% among Swedish-born women [41]. Of particular relevance to the focus of the present study, women who had immigrated after age 30 were less likely to participate in screening [41]. Taken together with the increased mortality rate ratio among women born outside Sweden and that those who immigrated later are less likely to have Swedish language fluency, special outreach efforts are clearly needed. In the present study, the non-participants, due in all but one case to lack of Swedish language proficiency, were on the average seven years older than the study participants. Culturally-tailored intervention programs have long been shown to effectively increase screening for cervical cancer and other malignancies [46]. Overcoming the language barriers is critical and this requires allocation of resources to translate the relevant informational materials into a number of different languages [17].

The participants in the present study represent quite a unique cohort of women who through participation in cervical cancer screening were diagnosed and treated for high-grade CIN and who attended follow-up. As noted, the study participants were more highly educated and with higher incomes than the overall Swedish population. Sweden as a whole has an organized, population-based cervical cancer screening program, and the cervical cancer mortality rates are among the lowest in the EU [3]. For many countries in the EU, especially those with the highest mortality rates from cervical cancer, the participation in cervical cancer screening is extremely low (< 20%) or there is no public cervical cancer screening program in place [3]. We have pointed out that the vast majority of cervical cancer deaths occur in less developed regions of the world. In several African countries and Latin American countries, cervical cancer is still the leading cause of cancer deaths among women [2]. Thus, questions about the generalizability of the present findings can arise, especially outside Sweden.

Even among women who do attend cervical cancer screening programs in Sweden, on-time screening is often low [17, 42]. Competing needs that appear to be more urgent, including requirements for taking time from work, are frequently cited barriers to cancer screening among women [17, 22, 47, 48]. Moreover, postponing is often not recognized as non-attendance [42]. Especially in light of the newly developing guidelines [1, 5, 6, 34], it is essential to effectively convey accurate information to all women about their own cervical cancer risk, needed preventive and follow-up measures, together with the relevant specific knowledge. Concerted efforts are also needed to ensure that this knowledge is translated into adherence to the clinical recommendations. This is particularly vital for improving adherence to recommended follow-up among women, such as those in the present study, who are at increased risk for developing cervical cancer. Tailored prevention programs are urgently needed for women at high risk for cervical cancer. These should include oral and written information from health care professionals at the care site, together with clinical follow-up. Moreover, increasing knowledge regarding HPV and cervical cancer among health professionals is very important, at the time when Sweden and other countries are preparing to incorporate primary screening by HPV.

## Supporting information

S1 Appendix Dataset. (XLSX)
